# Supplementary material for: Exploring communication between parents and clinical teams following children’s heart surgery: a survey in the UK
Source: BMJ Paediatr Open. 2019 Mar 22;3(1):e000391. doi: 10.1136/bmjpo-2018-000391 (PMC6542420; doi:10.1136/bmjpo-2018-000391)
Supplement: Supplementary file 1 [file bmjpo-2018-000391supp001.pdf]

Cardiology in the Young 2016; Page 1 of 10 © Cambridge University Press, 2016. This is an Open Access article, distributed under the terms of the Creative Commons Attribution licence (http://creativecommons.org/licenses/by/4.0/), which permits unrestricted re-use, distribution, and reproduction in any medium, provided the original work is properly cited.
doi:10.1017/S1047951116001256

Original Article

Definition of important early morbidities related to paediatric cardiac surgery

Katherine L. Brown,[1] Christina Pagel,[2] Rhian Brimmell,[3] Kate Bull,[1] Peter Davis,[4] Rodney C. Franklin,[5] Aparna Hoskote,[1] Natasha Khan,[6] Warren Rodrigues,[7] Sara Thorne,[8] Liz Smith,[1] Linda Chigaru,[1] Martin Utley,[2] Jo Wray,[1] Victor Tsang,[1] Andrew Mclean[7]

[1]Cardiac, Critical Care and Respiratory Division, Great Ormond Street Hospital NHS Foundation Trust; [2]Clinical Operational Research Unit, University College London; [3]Department Paediatric Cardiology and Cardiac Surgery, Evelina London Children's Hospital, London; [4]Paediatric Intensive Care Unit, Bristol Royal Hospital for Children, Bristol; [5]Paediatric Cardiology Department, Royal Brompton and Harefield NHS Foundation Trust, London; [6]Department of Cardiac Surgery, Birmingham Children's Hospital, Birmingham; [7]Departments of Paediatric Intensive Care and Paediatric Cardiac Surgery, Royal Hospital for Children at Yorkhill, Glasgow; [8]Department of Cardiology, University Hospital Birmingham, Birmingham, United Kingdom

Abstract *Background:* Morbidity is defined as a state of being unhealthy or of experiencing an aspect of health that is "generally bad for you", and postoperative morbidity linked to paediatric cardiac surgery encompasses a range of conditions that may impact the patient and are potential targets for quality assurance. *Methods:* As part of a wider study, a multi-disciplinary group of professionals aimed to define a list of morbidities linked to paediatric cardiac surgery that was prioritised by a panel reflecting the views of both professionals from a range of disciplines and settings as well as parents and patients. *Results:* We present a set of definitions of morbidity for use in routine audit after paediatric cardiac surgery. These morbidities are ranked in priority order as acute neurological event, unplanned re-operation, feeding problems, the need for renal support, major adverse cardiac events or never events, extracorporeal life support, necrotising enterocolitis, surgical site of blood stream infection, and prolonged pleural effusion or chylothorax. It is recognised that more than one such morbidity may arise in the same patient and these are referred to as multiple morbidities, except in the case of extracorporeal life support, which is a stand-alone constellation of morbidity. *Conclusions:* It is feasible to define a range of paediatric cardiac surgical morbidities for use in routine audit that reflects the priorities of both professionals and parents. The impact of these morbidities on the patient and family will be explored prospectively as part of a wider ongoing, multi-centre study.

Keywords: Morbidity; complication; paediatric cardiac surgery; outcome

Received: 20 April 2016; Accepted: 6 July 2016

Background

Morbidity is defined as a state of being unhealthy or of experiencing an aspect of health that is "generally bad for you". Morbidity associated with paediatric cardiac surgery is illness or lack of health that has a temporal connection to such an operation, and as such may be regarded as an adverse outcome. The Society of Thoracic Surgeons Taskforce Subcommittee on Patient Safety has defined a range of unwanted events that may contribute to postoperative morbidity, including complications, adverse events, harm, medical error or injury, and near misses.[1] This Patient Safety Taskforce further noted that in the current era of falling mortality rates after paediatric cardiac surgery, improvement in healthcare as measured by reduction in adverse

Correspondence to: K. Brown, Cardiac, Critical Care and Respiratory Division, Great Ormond Street Hospital NHS Foundation Trust, Great Ormond Street, London WC1N 3JH, United Kingdom. Tel: +44 207 813 8180; Fax: +44 207 829 8673; E-mail: Katherine.Brown@gosh.nhs.uk

Downloaded from http://www.cambridge.org/core. IP address: 50.133.180.162, on 05 Oct 2016 at 13:05:59, subject to the Cambridge Core terms of use, available at http://www.cambridge.org/core/terms. http://dx.doi.org/10.1017/S1047951116001256

outcomes is more likely when unwanted events are acknowledged, measured, and responded to in terms of healthcare delivery[1].

The main focus of our study, which has a national setting in the United Kingdom, is upon early paediatric cardiac surgical morbidities that are considered potentially avoidable, reducible, or can be mitigated. This is important to achieve as children who experience prolonged hospitalisation with complications, which are occurrences associated with an intervention that represent a departure from the desired course of events and are linked to suboptimal outcome[1], are at greater risk of death[2,3] Furthermore, over the long term, children with specific heart conditions who experienced prolonged stays in hospital following surgery also developed higher levels of neurological disability.[4,5] Prolonged stays in hospital may be required when a patient takes longer to recover after surgery because of complications, but it is well recognised that prolonged hospitalisation may itself expose patients to the likelihood of further hospital-associated adverse events. A significant complication of paediatric cardiac surgery and mechanical circulatory support is linked to neurological disability in around 50% of cases[6] and may cost in excess £10,000/day to implement.[7,8]

Routine audit of postoperative mortality is well established in the United Kingdom via the National Congenital Heart Diseases Audit (NCHDA),[9] which has published centre-specific results of individual operations online since 2005. Stakeholders including children's heart surgery programmes, congenital heart patient support groups, and National Congenital Heart Diseases Audit share a goal of reporting morbidity, but acknowledge that to enable routine monitoring of morbidities approaches to data analysis and display must be developed alongside defining suitable measures for routine use. A series of detailed articles by professionals from the United States of America-based Multi-Societal Database Committee for Pediatric and Congenital Heart Disease profiled an extensive range of complications incorporating all organ systems.[10–17] The Society of Thoracic Surgery database selected a narrower range of defined major complications that were retrospectively available within the Registry, and demonstrated that rates varied from 1 to 38% with greater prevalence at increased procedural complexity.[18] A further study indicated that prospective monitoring of complications may lead to greater case ascertainment, and hence a perception of higher complication rates[19].

Views may differ between professionals and non-professionals over what exactly the term morbidity refers to, and which morbidity events are most important. A recent study showed differing perceptions and priorities between clinicians and patients regarding chronic obstructive pulmonary disease services and outcomes.[20] Focus groups and formal consensus methods have been used to elicit patient and carer perspectives and determine group priorities in many contexts.[21] The nominal group technique was successfully used among general practitioners to identify prioritised lists of quality markers for the management of children in general practice[21] and by kidney transplant patients in ranking outcomes by importance.[22] Our study, which aimed to identify the incidence and impact of important early morbidities following paediatric cardiac surgery,[23] has been undertaken within the context of the United Kingdom National Health Service. As is depicted in Figure 1, which displays our study methodology, we utilised information from a systematic review of the literature that screened 1169 publications, an online discussion forum between families of patients with CHD, and three focus groups with CHD families run by the patient and family support group Children's Heart Federation, to identify, as far as possible, the entire range of known morbidity events. A group representing individuals from a range of backgrounds – the "Selection Panel" – which comprised 15 people – three family representatives, three paediatric cardiac surgeons, two paediatric intensive care doctors, two paediatric cardiologists, two paediatricians, a paediatric intensive care nurse, a clinical nurse specialist, and a clinical psychologist with experience of working with children with CHD and their families – prioritised the possible morbidities using the nominal group technique and secret voting. Working in parallel alongside this "Selection Panel", a second group of professionals, referred to as the "Definition Panel" (see Acknowledgements), worked with the prioritised list of potential morbidities to both define and assess the practicality of measuring them in routine clinical practice. This article details the definitions of morbidity that the panel recommended.

Methods

Development of operational definitions for routine morbidity monitoring

Over 1 year, we convened two meetings of a surgical morbidity "Definition Panel" that included three paediatric cardiac surgeons, where one was the chair, three paediatric cardiologists, with one specialising in adult CHD, three paediatric intensive care specialists, and two children's heart disease nurses. The "Definition Panel" had the following goals:

- Establish diagnostic criteria that constitute the definition of each of the morbidities, as prioritised by the "Selection Panel".
- Define the measurement protocol for each of the morbidities, including any aspects that require

Downloaded from http:/www.cambridge.org/core. IP address: 50.133.180.162, on 05 Oct 2016 at 13:05:59, subject to the Cambridge Core terms of use, available at http:/www.cambridge.org/core/terms.
http://dx.doi.org/10.1017/S1047951116001256

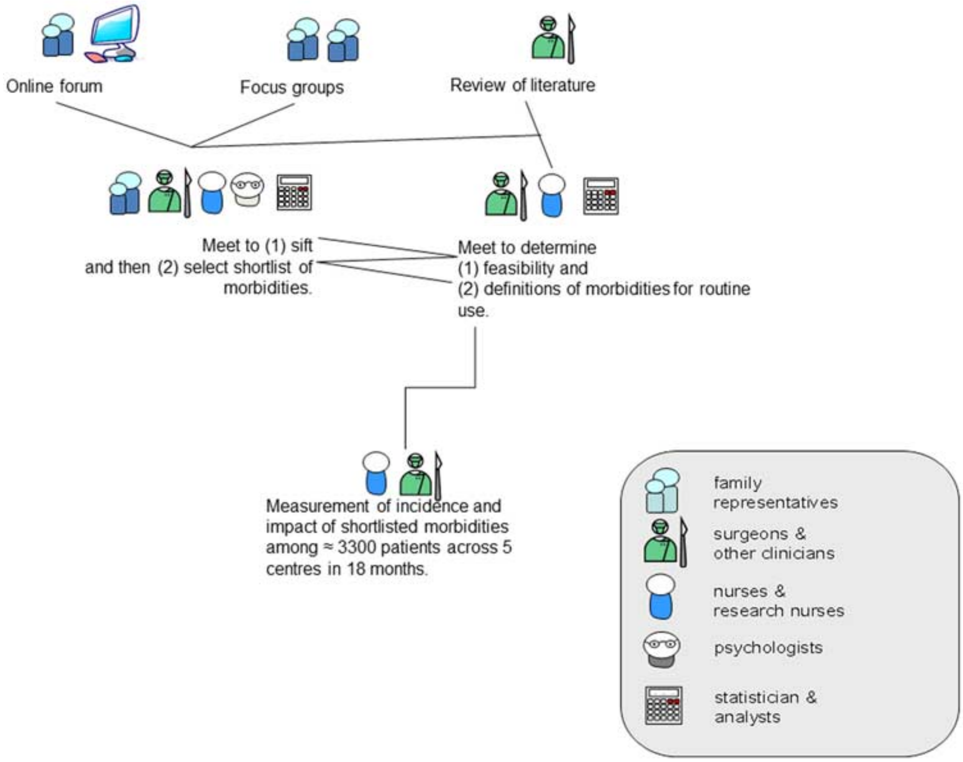

Figure 1.
The process that was followed for the selection of morbidities. A list of candidate morbidities was generated on the basis of a combination of systematic review of the literature, three focus groups with parents of children with CHD and young people with CHD, and an online discussion forum with CHD families. Morbidities were considered by a "Selection Panel" consisting of professionals from a range of backgrounds and lay people and were selected using the Nominal Group Technique and Secret Voting. The definitions of selected morbidities were undertaken by a group of United Kingdom-based specialist practitioners as listed in the report referred to as the "Definition Panel".

additional specialist input or alternatively surveillance outside the tertiary centre.
- Outline the minimum standards of the clinical pathway and necessary referrals and treatment for children who experience morbidities over the first 6 months after surgery.

This third part of the study drew upon information from the literature review and any relevant, established guidelines.[10–17,24]

In the first phase of the study, conducted through an initial face-to-face meeting followed by e-mail correspondence, the group provided the "Selection Panel" with views as to whether each candidate morbidity nominated by the first meeting of the "Selection Panel" was definable, measureable, and feasible to measure in routine practice, highlighting any additional issues identified in relation to each morbidity; one or two clinical leads were identified to take forward each of the individual shortlisted morbidities, utilising both e-mail and web-based interactions to develop each morbidity definition, reporting back at the second meeting of the definition group with an agreed package to sign off. Clinical leads consulted with other experts in the relevant field in order to optimise definitions and protocols where necessary. The protocols for identification, measurement, and management of shortlisted morbidities, including the timings of measurements, were designed for use in a multi-centre evaluation of morbidity incidence and impact, with suitability for routine use as a key requirement.

The morbidity definitions were implemented by a small group of nurses, intensive care doctors, and cardiothoracic surgeons within five children's heart centres based in the United Kingdom over a period of 2 months and used to prospectively record cases of morbidity as part of a wider research study.[23] During this time period, the definitions underwent further refinement and clarification in order to ensure that they were workable in the context of routine audit within the National Health Service.

Results

Morbidity definitions

Each definition is described in turn incorporating the timescale for identification of the morbidity, the definition, measurement protocol, and early

Downloaded from http:/www.cambridge.org/core. IP address: 50.133.180.162, on 05 Oct 2016 at 13:05:59, subject to the Cambridge Core terms of use, available at http:/www.cambridge.org/core/terms.
http://dx.doi.org/10.1017/S1047951116001256

management protocol in brief as described by the group. Morbidities are listed in priority order as determined by the selection panel.

Multiple morbidities and items not included

Within the context of the present study,[23] which aims to prospectively measure the incidence and impact of defined morbidity events, the approach to the number of morbidities in a given patient will be to identify single morbidity events as defined (1–9), extracorporeal life support morbidity events, which may incorporate further identified morbidities alongside, and multiple morbidities in instances where a patient has one or more morbidities excluding extracorporeal life support.

The "Selection Panel" further highlighted the importance of prolonged hospitalisation and poor communication between the treating team and the family, which they considered to be morbidities. It was noted by the definition panel that prolonged hospitalisation is linked to all post-procedural complications, and hence including length of stay as a morbidity would make measurement of the incidence of individual morbidities very challenging. Length of stay data including ventilation times, ICU length of stay, and hospital length of stay will be reviewed as part of the data analysis at the end of the study.

The "Definition Panel" considered that there was potential to define poor communication between the treating team and family in the future, but that it would necessarily involve asking parents about their experience in a way that would involve new data collection. The quality of communication between the treating team and the family has previously been assessed within the context of a patient satisfaction survey for all paediatric inpatients in England commissioned by the Care Quality Commission and undertaken by the organisation Picker Institute Europe.[25] The survey questions were formally developed using focus groups and were formally validated. The Picker Institute agreed to assist the definitions panel in identifying a short list of six questions to ask parents about communication and issued the research team with licence to allow our study to use these questions for patients recruited to a 6-month follow up sub-study to delineate this issue further. From a long list of 25 candidate questions from the Picker Questionnaire identified by the definition panel, Picker ran the following analysis:

- Frequency analysis to ascertain the percentage of missing data and the percentage of patients answering each of the possible responses.
- Inter-item correlation analysis and principal component analysis to identify questions that provided different dimensions of communication experience.

Picker then advised the Definition Panel on five to seven questions that could be asked of parents within 6 weeks of the patient's primary operation. The final questions chosen by the Definition Panel after discussion with Picker are as follows:

Q1. Did new members of staff treating your child introduce themselves?
Q2. Were you encouraged to be involved in decisions about your child's care and treatment?
Q3. Were you told different things by different people, which left you feeling confused?
Q4. Were the different members of staff caring for and treating your child aware of their medical history?
Q5. Before the operation or procedure, did a member of staff explain to you what would be done during the operation or procedure?
Q6. Did a member of staff tell you what to do or who to talk to if you were worried about your child when you got home?

We did not set a threshold for what defines "poor communication"; instead, we will explore the range of responses among control and case patients in our 6-month follow-up sub-study and possible associations with other clinical factors as part of a secondary data analysis.

Discussion

We present a list of consensus-based definitions of morbidities arising with paediatric cardiac surgery that have been designed for prospective audit. The prioritised and defined morbidities reflect a range of viewpoints and priorities, including those of both professionals and patients or parents. The professionals involved in our study represent tertiary, secondary, and primary care, and furthermore we have involvement in our study from at least one professional from every specialist paediatric cardiac surgical centre in the United Kingdom. We note that the list contains morbidities that were previously prioritised as "complications" by specialist professionals and included in a recent consensus-based statement from the United States of America-based Multi-Societal Database Committee for Pediatric and Congenital Heart Disease,[26] which are extracorporeal life support, renal support, pacemaker placement, diaphragm palsy, new permanent neurological deficit, and re-operation, but our list also contains further items not previously identified and prioritised, which are feeding problems, prolonged pleural effusion, and sepsis.

Challenges and limitations

In reaching these definitions certain challenges arose.

Consideration of pre-procedural factors. A major difficulty when contemplating the monitoring of

Downloaded from http:/www.cambridge.org/core. IP address: 50.133.180.162, on 05 Oct 2016 at 13:05:59, subject to the Cambridge Core terms of use, available at http:/www.cambridge.org/core/terms.
http://dx.doi.org/10.1017/S1047951116001256

morbidity following paediatric cardiac surgery is achieving a distinction between the morbidity that was present in the patient before the operation and the new morbidities that arose after surgery. It must be acknowledged that preoperative events such as existing congenital diagnoses and patient condition are inextricably linked to the postoperative journey,[3,5] and indeed both preoperative and postoperative events matter for the patient. Preoperative events may also potentially be subject to quality control – for example, the collapse of a neonate from late diagnosis of heart disease leads to higher rates of multiple organ failure[27] and may be averted by antenatal diagnosis and prospective management of the circulation.[28] Nonetheless, our focus is upon early outcomes after paediatric cardiac surgery and not on the entire care pathway; therefore, the definitions are designed to delineate postoperative events as clearly as possible. The delineation of new neurological morbidity in a postoperative patient may be challenging because of the inherent difficulties of assessing (in particular) small infants who may be critically ill. Prospective serial evaluation including preoperative and postoperative scans and detailed neurodevelopmental follow-up is ideal; however, this is not feasible within a United Kingdom National Health Service context, where cranial scans may only be undertaken on the basis of clinical indicators of suspected neurological injury, and hence our definition is pragmatic by necessity, although we hope that in the future it will be supplemented by enhanced methods of assessment.

Post-procedural timing. Conventionally, the time horizon linked to surgical complications has been considered as 30 days following the operation,[26] and for mortality outcomes Registries such as Society of Thoracic Surgery view the relevant time horizon as within the same operative hospitalisation or 30 days, whichever is longer.[29] For the majority of morbidity definitions, the time limit of either within 30 days or within the same hospitalisation was applied (see Table 1), based on what was considered most appropriate for the individual morbidity event. Certain morbidities, particularly those defined by the use of technology, such as renal support and extracorporeal life support, are only likely to occur within a hospitalisation, whereas others may occur at any time point over an operated child's lifespan – for example, re-operation, endocarditis, and feeding problems – and hence a time limit was placed accordingly in order to enhance the feasibility of postoperative audit, despite this time limit in some cases appearing arbitrary. It was noted that deep surgical site infection or mediastinitis, although always linked to cardiac surgery, may arise after discharge home and later than 30 days after surgery, and thus the timeline was extended for this morbidity.

Consistency and complexity of definitions. There are inherent practical difficulties with prospective audit of complex outcome measures; this is one reason for the historic focus on mortality as an outcome as this is much easier to measure than morbidity. For some morbidities, a treatment indicating the presence of morbidity was considered the better option rather than basing the diagnosis on clinical findings. This applies to the postoperative morbidities of renal failure, diaphragm paralysis, and feeding problems, for which postoperative renal support, the need for diaphragm plication, and technology-assisted feeding at discharge were selected as the most objective definitions available. A concern with using a treatment rather than a diagnosis as a measure of morbidity is that treating centres may initiate therapy at differing thresholds. During the course of our study, additional data items will be collected to explore the potential for such variation. As an example, practice patterns with respect to technology-assisted feeding in cardiac babies vary widely between geographic regions and diagnostic groups, and it is acknowledged that the audit of feeding problems at discharge rather than over time in outpatients may not capture the full picture.[30] For the case of extracorporeal life support, there is an inextricable link between the severe condition of patients requiring this therapy and the burden of the treatment itself,[5,26,31] and therefore this is reasonably widely accepted as a major morbidity after paediatric cardiac surgery by all stakeholder groups.[1] Moreover, considering the example of renal failure, given the complex inter-relationship between the patient's preoperative condition, which may incorporate renal dysfunction[32], their age, especially very young neonates as is common[27], their body mass index, which may be low in CHD, their postoperative condition, and measures of renal function, a definition involving a specified measure of renal function was considered to be impractical to define for routine use. Of note, it proved infeasible for the panel to agree a clear and usable definition of low cardiac output syndrome for use in routine audit.

Future steps

The definitions presented in Table 1 incorporate feedback from five United Kingdom paediatric cardiac surgical centres that have been using them prospectively for 5 months with paediatric cardiac surgery patients; however, we acknowledge that as yet the long-term practicalities involved in monitoring these morbidities are unclear. The next stage is to report on the morbidities for the purposes of quality

Downloaded from http:/www.cambridge.org/core. IP address: 50.133.180.162, on 05 Oct 2016 at 13:05:59, subject to the Cambridge Core terms of use, available at http:/www.cambridge.org/core/terms.
http://dx.doi.org/10.1017/S1047951116001256

Table 1. Morbidities with timescale for identification, definition, measurement protocol and minimum treatment protocol.

Morbidity	Timescale for identification	Definition	Measurement protocol (if additional to definition)	Minimum treatment protocol
Acute neurological event	Includes neurological morbidities that, based on best clinical judgement, arose as *new* findings around the time of surgery that were detected within the same hospitalisation as the surgery. It is recognised that in certain circumstances such as where a child is very sick on life support, pre-procedure assessment is challenging, in these circumstances as full an evaluation as possible to be completed, incorporating serial assessments over time	Neurological events including: seizure, abnormal movement (includes choreiform or athetoid), focal neurological deficit (includes hemiplegia and monoplegia), intracranial haemorrhage, stroke, brain death, reversible ischaemic neurological dysfunction, hypoxic ischaemic encephalopathy, spinal cord ischaemia, basal ganglia damage, or brain stem injury (includes abnormal cough or gag reflex)[38]	Includes new abnormality in any of the following:<br>•Electroencephalogram.<br>•Brain scan (either CT or MRI)<br>•Clinical evaluation<br>(seizures or movement disorder, focal neurological signs, generalised neurological signs, altered conscious level including even brain death)	The treatment protocol is variable depending on the type of neuro-morbidity.<br>Specialist consultation with a neurologist, a full evaluation of any brain injury and neuro-developmental follow-up would be a minimum
Unplanned re-operation or re-intervention	Unplanned re-interventions are procedures outside the expected patient pathway, which may be undertaken at any time from the start of the postoperative admission up until 30 days following the primary operation. Additional procedures or revisions undertaken within the primary trip to the operating theatre (incorporating return onto cardiopulmonary bypass) are not included in the definition of re-operation	Unplanned re-interventions include procedures that were not intended during the planning phase, follow an initial primary cardiac surgery and result in "substantive alteration to heart" incorporating cardiac bypass, cardiac non-bypass, pacemaker placement, interventional catheterisations, and also diaphragm plications (which are not related to the heart itself). The definition does not include support or other non-cardiac surgery procedures	Unplanned return to the operating room or cardiac catheter laboratory within 30 days<br>(excludes interventional catheters that were planned preoperatively; excluding delayed chest closure, excluding procedures for bleeding)<br>(Includes diaphragm plication and insertion of pacemaker for surgically acquired arrhythmia)	Not applicable. The minimal assessment is cardiovascular evaluation of the repair with echocardiography and tolerance of weaning from life supports
Feeding problems	A diagnosis of postoperative feeding problems should be considered during recovery after surgery and before discharge from the specialist centre either to home or to secondary care if the child is unable to feed normally. The goal is detection of feeding problems which are new post-surgery, and it is recognised that this may be challenging where a child was not fed preoperatively for cardiac reasons as feeding ability will not have been assessed objectively	A child may fail to feed normally following paediatric cardiac surgery for a range of reasons including gastro-oesophageal reflux, vocal cord paralysis, oral-motor dysfunction, oral aversion, and neurologic impairment.[31] If for any of these reasons a child is not able to orally feed or completely orally feed and is tube dependent at discharge from the tertiary centre or at 30 days (if he or she is otherwise clinically stable enough to feed at that time point), then a postoperative feeding problem will be diagnosed	The requirement for any feeding support. Includes via the intravenous route or via an enteral tube.<br>Excludes feeding support that was present to treat a primary problem diagnosed before the surgery, feeding support related to an episode of necrotising enterocolitis, and feeding support because the child dislikes a special diet	Treatment includes assessment by the dietician, speech and language therapist and of the patient's weight. Progress with feeding should be monitored by the clinical care team responsible at each stage of the journey
Need for renal replacement therapy	Includes renal replacement therapy when initiated as a new support at any time from the start of the postoperative admission to ICU up until 30 days following the primary operation	The child requires renal replacement therapy (peritoneal dialysis or haemofiltration) for renal failure (oligo-anuria of >0.5 ml/kg/hour and elevated creatinine level for age) and or fluid overload. In patients where renal support is required alongside extracorporeal life support, the primary morbidity is viewed as extracorporeal life support	The measurement protocol is simply the presence of (new) renal support.<br>(Excludes renal support on extracorporeal life support). Data on renal biochemistry and urine output will be collected	Instigation of effective renal replacement therapy.<br>If recovery of kidney function does not occur within 3–5 weeks then consultation with paediatric renal physician is required

Downloaded from http:/www.cambridge.org/core. IP address: 50.133.180.162, on 05 Oct 2016 at 13:05:59, subject to the Cambridge Core terms of use, available at http:/www.cambridge.org/core/terms. http://dx.doi.org/10.1017/S1047951116001256

Downloaded from http:/www.cambridge.org/core. IP address: 50.133.180.162, on 05 Oct 2016 at 13:05:59, subject to the Cambridge Core terms of use, available at http:/www.cambridge.org/core/terms. http://dx.doi.org/10.1017/S1047951116001256

Major adverse cardiac events or never events	Events within this morbidity may be identified during the tertiary hospital stay (either ward or ICU) following the primary surgery	This morbidity includes<br>cardiac arrest, where the child receives any chest compressions or defibrillation<br>Chest re-opening on the ICU or ward for any reason<br>Major haemorrhage in the ICU following surgery<br>A "Never Event" applicable to paediatric cardiac surgery as selected from the "Never Events" list published for National Health Service for 2015[39] (Including wrong site or wrong patient surgery, wrong prosthesis surgery, retained foreign object post-procedure, wrong route administration of medication, transfusion or transplantation of main red cell group incompatible blood components or organs, misplaced naso-gastric or oro-gastric tubes<br>Tissue injury to limb or vital organ such as perforated viscus or ischaemic limb injury	Major haemorrhage is defined as bleeding >10 ml/kg/hour on ICU for 2 consecutive hours.<br>A "Never Event" includes the events listed plus harm to the patient, for example: if a naso-gastric tube is misplaced, detected and removed in a timely manner before any harm is done then this is not a "Never Event". Conversely, if the misplaced naso-gastric tube is not noted, and feed is given into the bronchus, then this is a "Never Event"	All events will results in immediate treatment as part of current practice
Extracorporeal life support	Extracorporeal life support following surgery and before discharge from the tertiary hospital, including the rare cases when a child was on extracorporeal life support before surgery	This morbidity is defined by the presence of an extracorporeal life support system connected to the patient following the operation, whether it was placed in the operating theatre or in the ICU, and whether the indication was cardiac arrest, low cardiac output state, poor cardiac function, arrhythmia, residual or recurrent cardiac lesion, pulmonary including pulmonary hypertension or sepsis	It is recognised that children on extracorporeal life support following paediatric cardiac surgery have high rates of other complications including renal support, bleeding, sepsis, sternal re-opening, and cardiac arrest.[40] Where such complications arise as part of extracorporeal life support, the morbidity is defined as extracorporeal life support	The morbidity is in fact a treatment modality offered so this is not applicable. Centres offering extracorporeal life support follow protocols based on those provided by the extracorporeal life support organisation[24]
Necrotising enterocolitis	Necrotising enterocolitis as a new diagnosis from after surgery until discharge from the tertiary hospital	Necrotising enterocolitis class 1a or 1b,[41] which incorporates babies with systemic signs of inflammation and abdominal clinical signs such as distension or larger than normal gastric aspirates or mild rectal bleeding but no radiological changes are included, if a general surgery specialist has seen the child and commenced a course of intravenous antibiotics and parenteral nutrition for five to seven days. Cases of severe necrotising enterocolitis with radiological signs systemic instability and bowel perforation are also included	Data in respect of systemic clinical signs, intestinal signs, and radiology will be collected, as well as the treatments deployed, thus enabling the necrotising enterocolitis diagnosis to be graded between 1a and 3b[41]	Consultation with general surgery and further management in respect of antibiotics, nutrition, radiological investigation, and surgical intervention
Surgical site infection and blood stream infection	Surgical site and blood stream infections diagnosed within the hospital admission following surgery or following readmission to the same unit during postoperative recovery, where the treating clinical team assesses the infection to be linked to the recent operation. It is noted that mediastinitis may be detected more	Deep surgical site infection and/or mediastinitis includes any infection of an incised wound that undergoes any re-intervention by a surgeon (such as opening of the wound, vacuum dressing), mediastinitis and false aneurysm, independent of culture positivity.<br>Blood stream infection includes both catheter related and non-catheter related. Cases have	Deep surgical site infection excludes superficial site infection managed without a surgeon's reoperation by conventional nurse dressing only, even if the wound heals by secondary intention	The minimum treatment protocol consists of antibiotics based on organism and sensitivities, and where relevant the removal of the line. Surgical intervention may be required for deep surgical site and in some cases of endocarditis. Both conditions require prolonged antibiotic therapy

Table 1. *Continued*

Morbidity	Timescale for identification	Definition	Measurement protocol (if additional to definition)	Minimum treatment protocol
	than 30 days after cardiac surgery,[42] hence this time cut-off is not applicable	systemic signs of infection, a positive culture not judged to be a contaminant, and in the case of line related a catheter in place with positive cultures from the line or from the line tip when removed<br>Endocarditis based on clinical, imaging or culture evidence judged to be diagnostic of endothelial/endocardial infection and its sequelae cardiac or extra-cardiac		
Prolonged pleural effusion or chylothorax	Prolonged pleural effusion is a post-procedural effusion with duration greater than 10 days. Chylothorax is diagnosed from after surgery until discharge from the tertiary hospital	Either a chylous pleural effusion or significant chylous pericardial effusion or significant chylous ascites or a prolonged non-chylous effusion that necessitates thoracic drainage at least 10 days following index cardiac surgery	Chylous effusions are characterised by milky appearance and a pleural fluid white blood cell count of >1000 cells/μl with lymphocytes >80%.[32] If the child is on normal feeds the triglyceride level in the pleural fluid will be >1.1 mmol/L or the ratio between the pleural triglyceride level and the serum triglyceride level will exceed 1	Diet consisting of medium-chain triglycerides or low fat for chylothorax. On a patient-by-patient basis other treatments include parenteral nutrition, octreotide infusion, intervention for venous obstruction thoracic duct ligation, and pleuradhesis

Downloaded from http://www.cambridge.org/core. IP address: 50.133.180.162, on 05 Oct 2016 at 13:05:59, subject to the Cambridge Core terms of use, available at http://www.cambridge.org/core/terms. http://dx.doi.org/10.1017/S1047951116001256

assurance and to assess their impact on patients and families with formal prospective analysis. We note that analytical and graphical methods for the timely reporting of risk-adjusted mortality outcomes for the purposes of quality improvement are well established in adult cardiac surgery practice[33] and have been developed by members of our research group for paediatric cardiac surgery;[34] two single-centre studies have attempted to generate an aggregate "Morbidity Index" by assigning subjective weights to postoperative complications,[35,36] and the Society for Thoracic Surgeon group have attempted a similar "Morbidity Score".[18] Condensing diverse morbidities into a single score loses information, and recent work on using graphical methods to routinely monitor a range of morbidities highlighted the complexity of graphically summarising multiple morbidities[19] (see also commentary by Utley et al[37]). We intend to report on the incidence and impact of the defined morbidities over the next few months: the findings of these studies will inform future data collection for national audit in the United Kingdom.

Acknowledgements

The authors acknowledge the work done by the research teams based in the paediatric cardiac surgery and ICUs at Great Ormond Street Hospital, Evelina London, Birmingham Children's Hospital, Bristol Children's Hospital, and The Royal Hospital for Children in Glasgow who have implemented these definitions and provided valuable feedback. The Definition Panel comprised the following individuals with specialist skills as listed: R.B., Evelina Children's Hospital, paediatric intensive care nurse and data expert. Dr K.B., Great Ormond Street Hospital, paediatric cardiologist and family liaison officer. Dr P.D., Bristol Royal Hospital for Children, paediatric intensive care consultant. Dr R.F., Royal Brompton Hospital, paediatric cardiologist. Dr A.H., Great Ormond Street Hospital, paediatric intensive care consultant. Ms N.K., Birmingham Children's Hospital, paediatric cardiac surgeon. Mr A.M., Royal Hospital for Children, Glasgow, paediatric cardiac surgeon and Chair. Dr W.R., Royal Hospital for Children, Glasgow, paediatric intensive care consultant. L.S., Great Ormond Street Hospital, intensive care nurse. Dr S.T., University Hospital Birmingham, cardiologist.

Financial Support

This project was funded by the National Institute for Health Research Health Services and Delivery Research Programme (Project No. 12/5005/06). K.B., L.S., A.H., J.W., L.C., and V.T. were supported by the National Institute for Health Research Biomedical Research Centre at Great Ormond Street Hospital for Children NHS Foundation Trust and University College London.

Conflicts of Interest

Dr K.B., Dr R.F. and Mr A.M. serve on the steering committee of the National Congenital Heart Diseases Audit (NCHDA).

Ethical Standards

The paediatric cardiac surgery morbidity study was granted ethical approval by London City Road Research Ethics Committee (REC) on 8th November 2013 and the REC number is 14-LO-1442.

References

1. Jacobs JP, Benavidez OJ, Bacha EA, Walters HL, Jacobs ML. The nomenclature of safety and quality of care for patients with congenital cardiac disease: a report of the Society of Thoracic Surgeons Congenital Database Taskforce Subcommittee on Patient Safety. Cardiol Young 2008; 18 (Suppl 2): 81–91.
2. Pasquali SK, Li JS, Burstein DS, et al. Association of center volume with mortality and complications in pediatric heart surgery. Pediatrics 2012; 129: e370–e376.
3. Brown KL, Ridout DA, Goldman AP, Hoskote A, Penny DJ. Risk factors for long intensive care unit stay after cardiopulmonary bypass in children. Crit Care Med 2003; 31: 28–33.
4. Bellinger DC, Wypij D, duPlessis AJ, et al. Neurodevelopmental status at eight years in children with dextro-transposition of the great arteries: the Boston Circulatory Arrest Trial. J Thorac Cardiovasc Surg 2003; 126: 1385–1396.
5. Newburger JW, Sleeper LA, Bellinger DC, et al. Early developmental outcome in children with hypoplastic left heart syndrome and related anomalies: the single ventricle reconstruction trial. Circulation 2012; 125: 2081–2091.
6. Lequier L, Joffe AR, Robertson CM, et al. Two-year survival, mental, and motor outcomes after cardiac extracorporeal life support at less than five years of age. J Thorac Cardiovasc Surg 2008; 136: 976–983 e3.
7. Mahle WT, Forbess JM, Kirshbom PM, Cuadrado AR, Simsic JM, Kanter KR. Cost-utility analysis of salvage cardiac extracorporeal membrane oxygenation in children. J Thorac Cardiovasc Surg 2005; 129: 1084–1090.
8. Brown KL, Wray J, Wood TL, Mc Mahon AM, Burch M, Cairns J. Cost utility evaluation of extracorporeal membrane oxygenation as a bridge to transplant for children with end-stage heart failure due to dilated cardiomyopathy. J Heart Lung Transplant 2009; 28: 32–38.
9. NICOR. NICOR: National Institute for Cardiovascular Outcomes Research: Congenital Heart Diseases website. UCL London UK: University College London; 2015. Retrieved 15 January 2015 from http://www.nicor4.nicor.org.uk/CHD
10. Cooper DS, Jacobs JP, Chai PJ, et al. Pulmonary complications associated with the treatment of patients with congenital cardiac disease: consensus definitions from the Multi-Societal Database Committee for Pediatric and Congenital Heart Disease. Cardiol Young 2008; 18 (Suppl 2): 215–221.
11. Bacha EA, Cooper D, Thiagarajan R, et al. Cardiac complications associated with the treatment of patients with congenital cardiac disease: consensus definitions from the Multi-Societal Database Committee for Pediatric and Congenital Heart Disease. Cardiol Young 2008; 18 (Suppl 2): 196–201.

Downloaded from http:/www.cambridge.org/core. IP address: 50.133.180.162, on 05 Oct 2016 at 13:05:59, subject to the Cambridge Core terms of use, available at http:/www.cambridge.org/core/terms.
http://dx.doi.org/10.1017/S1047951116001256

12. Bird GL, Jeffries HE, Licht DJ, et al. Neurological complications associated with the treatment of patients with congenital cardiac disease: consensus definitions from the Multi-Societal Database Committee for Pediatric and Congenital Heart Disease. Cardiol Young 2008; 18 (Suppl 2): 234–239.
13. Checchia PA, Karamlou T, Maruszewski B, Ohye RG, Bronicki R, Dodge-Khatami A. Haematological and infectious complications associated with the treatment of patients with congenital cardiac disease: consensus definitions from the Multi-Societal Database Committee for Pediatric and Congenital Heart Disease. Cardiol Young 2008; 18 (Suppl 2): 226–233.
14. Deal BJ, Mavroudis C, Jacobs JP, Gevitz M, Backer CL. Arrhythmic complications associated with the treatment of patients with congenital cardiac disease: consensus definitions from the Multi-Societal Database Committee for Pediatric and Congenital Heart Disease. Cardiol Young 2008; 18 (Suppl 2): 202–205.
15. Dickerson H, Cooper DS, Checchia PA, Nelson DP. Endocrinal complications associated with the treatment of patients with congenital cardiac disease: consensus definitions from the Multi-Societal Database Committee for Pediatric and Congenital Heart Disease. Cardiol Young 2008; 18 (Suppl 2): 256–264.
16. Ghanayem NS, Dearani JA, Welke KF, Beland MJ, Shen I, Ebels T. Gastrointestinal complications associated with the treatment of patients with congenital cardiac disease: consensus definitions from the Multi-Societal Database Committee for Pediatric and Congenital Heart Disease. Cardiol Young 2008; 18 (Suppl 2): 240–244.
17. Jacobs JP. Introduction – databases and the assessment of complications associated with the treatment of patients with congenital cardiac disease. Cardiol Young 2008; 18 (Suppl 2): 1–37.
18. Jacobs ML, O'Brien SM, Jacobs JP, et al. An empirically based tool for analyzing morbidity associated with operations for congenital heart disease. J Thorac Cardiovasc Surg 2013; 145: 1046–1057 e1.
19. Belliveau D, Burton HJ, O'Blenes SB, Warren AE, Hancock Friesen CL. Real-time complication monitoring in pediatric cardiac surgery. Ann Thorac Surg 2012; 94: 1596–1602.
20. Murphy MK, Black NA, Lamping DL, et al. Consensus development methods, and their use in clinical guideline development. Health Technol Assess 1998; 2: i–iv; 1–88.
21. Gill PJ, Hewitson P, Peile E, Harnden A. Prioritizing areas for quality marker development in children in UK general practice: extending the use of the nominal group technique. Fam Pract 2012; 29: 567–575.
22. Howell M, Tong A, Wong G, Craig JC, Howard K. Important outcomes for kidney transplant recipients: a nominal group and qualitative study. Am J Kidney Dis 2012; 60: 186–196.
23. National Institutes of Health Research. HS&DR – 12/5005/06: selection, definition and evaluation of important early morbidities associated with paediatric cardiac surgery. Project Portfolio 2014. 2014. Retrieved April 6, 2016 from http://www.nets.nihr.ac.uk/projects/hsdr/12500506
24. Centers for Disease Control and Prevention (CDC). Surgical Site Infection (SSI) Event, In: Gov U (ed.). *Procedure Associated Module (SSI)*. Centers for Disease Control and Prevention (CDC), Atlanta, GA. 2015: 1–26.
25. Europe P. Paediatric Inpatient Survey – Final. 2014 Retrieved December, 2014 from http://www.pickereurope.org/wp-content/uploads/2014/10/Paediatrics_inpatient_survey_FINAL.pdf
26. Jacobs JP, Jacobs ML, Austin EH, 3rd, et al. Quality measures for congenital and pediatric cardiac surgery. World J Pediatr Congenit Heart Surg 2012; 3: 32–47.
27. Brown KL, Ridout DA, Hoskote A, Verhulst L, Ricci M, Bull C. Delayed diagnosis of congenital heart disease worsens preoperative condition and outcome of surgery in neonates. Heart 2006; 92: 1298–1302.
28. Sivarajan V, Penny DJ, Filan P, Brizard C, Shekerdemian LS. Impact of antenatal diagnosis of hypoplastic left heart syndrome on the clinical presentation and surgical outcomes: the Australian experience. J Paediatr Child Health 2009; 45: 112–117.
29. Clarke DR, Breen LS, Jacobs ML, et al. Verification of data in congenital cardiac surgery. Cardiol Young 2008; 18 (Suppl 2): 177–187.
30. Medoff-Cooper B, Ravishankar C. Nutrition and growth in congenital heart disease: a challenge in children. Curr Opin Cardiol 2013; 28: 122–129.
31. Medoff-Cooper B, Naim M, Torowicz D, Mott A. Feeding, growth, and nutrition in children with congenitally malformed hearts. Cardiol Young 2010; 20 (Suppl 3): 149–153.
32. Zuluaga MT. Chylothorax after surgery for congenital heart disease. Curr Opin Pediatr 2012; 24: 291–294.
33. Sherlaw-Johnson C, Gallivan S, Treasure T, Nashef SA. Computer tools to assist the monitoring of outcomes in surgery. Eur J Cardiothorac Surg 2004; 26: 1032–1036.
34. Tsang VT, Brown KL, Synnergren MJ, et al. Monitoring risk-adjusted outcomes in congenital heart surgery: does the appropriateness of a risk model change with time? Ann Thorac Surg 2009; 87: 584–587.
35. Bojan M, Gerelli S, Gioanni S, Pouard P, Vouhe P. Evaluation of a new tool for morbidity assessment in congenital cardiac surgery. Ann Thorac Surg 2011; 92: 2200–2204.
36. Stoica S, Carpenter E, Campbell D, et al. Morbidity of the arterial switch operation. Ann Thorac Surg 2012; 93: 1977–1983.
37. Utley M, Brown K, Tsang V. Invited commentary. Ann Thorac Surg 2012; 94: 1602–1603.
38. Dominguez TE, Wernovsky G, Gaynor JW. Cause and prevention of central nervous system injury in neonates undergoing cardiac surgery. Semin Thorac Cardiovasc Surg 2007; 19: 269–277.
39. NHS. Never-events. Patient Safety 2015. Patient Safety Guideline. 2015.Retrieved August 10, 2015 from. http://www.england.nhs.uk/ourwork/patientsafety/never-events/
40. Chaturvedi RR, Macrae D, Brown KL, et al. Cardiac ECMO for biventricular hearts after paediatric open heart surgery. Heart 2004; 90: 545–551.
41. McElhinney DB, Hedrick HL, Bush DM, et al. Necrotizing enterocolitis in neonates with congenital heart disease: risk factors and outcomes. Pediatrics 2000; 106: 1080–1087.
42. Sohn AH, Schwartz JM, Yang KY, Jarvis WR, Guglielmo BJ, Weintrub PS. Risk factors and risk adjustment for surgical site infections in pediatric cardiothoracic surgery patients. Am J Infect Control 2010; 38: 706–710.

Downloaded from http:/www.cambridge.org/core. IP address: 50.133.180.162, on 05 Oct 2016 at 13:05:59, subject to the Cambridge Core terms of use, available at http:/www.cambridge.org/core/terms.
http://dx.doi.org/10.1017/S1047951116001256
